# Effective School Leadership for Supporting Students’ Mental Health: Findings from a Narrative Literature Review

**DOI:** 10.3390/bs15010036

**Published:** 2025-01-01

**Authors:** Brian P. Daly, Annie Resnikoff, Shannon Litke

**Affiliations:** Department of Psychological and Brain Sciences, Drexel University, 3141 Chestnut Street, Philadelphia, PA 19104, USA; ar3767@drexel.edu (A.R.); sgl46@drexel.edu (S.L.)

**Keywords:** leadership, school mental health, leadership preparation, professional development

## Abstract

There is a compelling literature base in the field of education that highlights how school leaders are crucial to improving academic processes and outcomes, including instruction and raising student achievement. Research has also demonstrated that effective school leaders exhibit behaviors aligned with promoting the core issues of teaching, learning, and school improvement. Less well-known is what science says about the effectiveness of professional development and leadership preparation programs for developing the competencies needed for addressing the diverse mental health needs of students. Better understanding the science is important as school leaders are increasingly tasked with connecting leadership behaviors to students’ social and emotional outcomes, and these leaders play a large role in whether, and to what extent, mental health promotion and prevention are effectively implemented in school settings. Drawing from diverse literature bases of education and psychology, the primary objective of this narrative literature review is to determine and define effective leadership behaviors, skills, and competencies in the context of implementing school mental health programs and strategies. The secondary objective is to summarize the existing research examining leadership preparation and professional development programs that promote effective leadership practices and highlight examples of leadership programs focused on enhancing school mental health.

## 1. Introduction

It is widely acknowledged that effective school leadership is essential if schools are to fulfill their mission by supporting students to achieve their educational goals and personal development (Sahlin, 2023). The roles and responsibilities associated with being a school leader (e.g., principal or assistant principal) have grown increasingly complex and now include serving a variety of diverse stakeholders with different agendas and needs, including students, parents, teachers, superintendents, and more. These new levels of complexity highlight the importance of future school leaders participating in leadership preparation programs as well as more established school leaders engaging in ongoing professional development. Most of the research on these leadership preparation and professional development programs has understandably focused on providing the necessary skills and competencies for leaders to have a meaningful impact on student learning and academic outcomes. Although not exclusive, school leaders generally influence these learning and academic outcomes indirectly through the work of their teachers (Robinson & Gray, 2019). For example, there is evidence to suggest that the most effective and high-quality school leaders achieve this impact by setting directions, developing people, and developing the right organizational conditions for effective work (Leithwood et al., 2004; Young et al., 2017). Therefore, leadership preparation and development programs focused on student learning outcomes have often sought to promote capacities associated with helping leaders engage in the following behaviors: (1) develop plans that all stakeholders understand, including establishing high expectations and then tracking progress and performance with data; (2) teach and train leaders how to provide the necessary supports and resources for teachers to be successful in the school; and (3) align incentives within the organization that facilitates the conditions for impactful teaching and student learning (Young et al., 2017).

While previous school leadership research and leadership program development mainly focused on how leaders can, directly and indirectly, support teachers to improve student academic outcomes, there is increasing recognition of the importance of effectively leading all school personnel, including counselors and mental health professionals, to ensure students’ mental health and well-being and the provision of equitable access to support for all students (Day et al., 2016; Sammons et al., 2016). In fact, it has been argued that addressing student socio-emotional and mental health issues represents a key and, potentially, the most complex challenge for school leaders (Adams & Olsen, 2017). This challenge was significant prior to 2020 but has only increased in the wake of the COVID-19 pandemic. For example, findings from large-scale studies that collected data on children between the pre- and post-shutdown periods reveal significant elevations in children’s mental health challenges in the post-shutdown period, including in their internalizing behaviors such as symptoms of depression and anxiety (Newlove-Delgado et al., 2021; Westrupp et al., 2020). Additional smaller studies documented changes in children’s pre- and post-shutdown outcomes, including increases in children’s dysregulated and externalizing behaviors and decreases in children’s adaptive behaviors (Hanno et al., 2021).

Schools are the most common setting for the delivery of mental health services to youth in the USA (Costello et al., 2014; Langer et al., 2015); therefore, a central responsibility of school leadership is to provide the necessary structures and sufficient levels of support for student mental health. Parker and colleagues (Parker et al., 2018) argued that the success of school mental health programs is inextricably linked with successful school leadership and studies have affirmed the pivotal role of the school leader in supporting student mental health by offering effective prevention and intervention activities in the school (see, for instance, Anyon et al., 2016; Forman et al., 2008; Short, 2016). It is surprising, therefore, that the literature on school leadership and school mental health is not more well developed given the consensus among practitioners and scholars that enacting comprehensive, coordinated mental health services begins with strong leadership.

Education reform initiatives over recent decades require that school leaders connect leadership behaviors to students’ social and emotional outcomes (Papa, 2018). However, the educational reform initiatives do not adequately detail or provide specifics about what comprises effective or “strong” leadership behaviors, skills, and competencies in the context of promoting and supporting student mental health. In this narrative literature review that included studies from the fields of education and psychology, we asked and sought to address the following question: Within the existing scholarship, what are effective leadership behaviors, skills, and competencies of school leaders? When applied to implementing school mental health programs and strategies? We also sought to determine existing models and best practice leadership development programs and then offer insights and suggestions on how these practices can be connected to school mental health promotion that is responsive to the diverse and often complex mental health needs of students.

For this narrative literature review, the following psychology and education research databases were searched because of their relevance to fields associated with leadership, schools, and student mental health: Scopus, Web of Science, PubMed, ERIC, and PsychInfo. Keywords in the search included: ‘school leadership’, ‘school principal’, ‘school leader’, ‘professional development’, ‘leadership preparation’, ‘leadership behaviors’, ‘leadership skills’, ‘leadership competencies’, ‘school mental health’, or ‘school mental health program’. Inclusion criteria were scientific journal articles, books, and book chapters written in English with a timeline between January 2000 and up to July 2024. Exclusion criteria were unpublished research, comments, editorials, master’s theses or dissertations, and non-English publications.

The narrative literature review revealed few studies, and no meta-analyses, that specifically addressed effective leadership behaviors in the context of implementing school mental health programs. This review, therefore, sought to make a unique contribution by reviewing leadership behaviors, skills, and competencies for a range of school-related needs and highlighting connections to how similar skills and competencies can support effective school leadership of mental health programs and associated personnel such as mental health clinicians tasked with supporting student mental health and well-being (see Table S1).

### 1.1. Individual Leadership Behaviors

There is a well-established literature base that highlights factors such as time management, influence, decision-making, commitment, and communication as essential for educational leadership effectiveness (Sun et al., 2014). More recently, as educational leaders are becoming tasked with supporting the mental health of all students (Rowling, 2009), there has been a shift toward emphasizing additional factors in leadership development programs that can promote social and emotional competencies of school leaders. Social and emotional competencies fall under the larger umbrella of emotional intelligence (EI), a construct proposed by Mayer and Salovey (1997) with four interrelated abilities: (1) perception/expression of emotion, (2) use of emotion to facilitate thinking, (3) understanding of emotion, and (4) management of emotion in oneself and others. EI is typically broken down into four core competencies that include self-awareness, self-management, social awareness, and relationship management. Parker and colleagues (Parker et al., 2018) argue the empirical link between EI and successful leadership can best be accounted for by motivational factors, cognitive factors, and interpersonal factors. EI is thought to impact successful work performance by enabling a person “to demonstrate intelligent use of their emotions in managing themselves and working with others to be effective at work” (Boyatzis et al., 2000, p. 2). However, as described below in the studies reviewed, the evidence supporting the impact of EI is scant, and available study findings are mixed at best.

As applied to the school setting, the theory would suggest that leaders high in EI are likely to improve school climate and school culture and model effective social and emotional competencies for school personnel who are more directly involved in the implementation of school mental health promotion, prevention, and intervention activities. Findings from a recent systematic review that examined the relationship between EI and leadership in school leaders revealed that EI is key for effective leadership and the most frequently used skills/and competencies are self-awareness, self-management, and empathy (Gómez-Leal et al., 2021). Several studies of school principals, vice-principals, and superintendents in Canada found that high-performing leaders, as compared to average or below-average leaders, demonstrated higher scores of total EI and the dimensions of intrapersonal, interpersonal, adaptability, and stress management as measured by the Emotional Quotient Inventory (Bar-On, 1997; Stone et al., 2005, 2008). In contrast, findings from a quasi-experimental pilot study that evaluated the effectiveness of a post-graduate development program for aspiring school leaders that incorporated activities to promote social and EI revealed no statistical significance in the tested variables after one semester (Sánchez-Núñez et al., 2015). When these same competencies were evaluated two years post-intervention, significant findings were demonstrated for the student candidates’ self-reported competencies directly related to leadership. However, the means of observer raters’ scores reflecting actual implementation or objective performance did not reveal statistically significant differences, suggesting mixed results for this leadership development program.

Findings from a multi-year program that focused on promoting and developing EI in school leaders also produced mixed and nuanced results (Keefer et al., 2014). For example, examination of pre- and post-means revealed no significant changes in EI abilities for all participants (*N* = 83). However, those with low EI abilities at baseline demonstrated the greatest improvement relative to those with high EI abilities at baseline. The authors interpreted the findings to suggest the program was the most impactful in improving EI abilities for those participants who were most in need. Strengths of the exploratory study included facilitators who previously served as school leaders and adequate dosage (2 years). Limitations of the study included a high drop-out rate (64%), attributed to the length of the program and a population of leaders who frequently change school districts. Taken together, despite the hypothesized link between EI and successful leadership and while some preservice and in-service school leadership programs have explicitly focused on improving EI skills and competencies, very few well-controlled studies have been conducted to determine short- or long-term impact (Gómez-Leal et al., 2021; Sánchez-Núñez et al., 2015). Beyond individual skills and competencies associated with leadership, there is an emerging area of focus in the literature on organizational leadership behaviors, particularly as they relate to successful implementation efforts.

### 1.2. Organizational Leadership Behaviors

According to Eriksson and colleagues (2017), school leadership for mental health promotion involves ‘leadership that is concerned with creating a culture for health promoting workplaces and values to inspire and motivate the employees to participate in such a development’ (Eriksson et al., 2017). Visible and proactive support from senior leadership in schools is essential to developing a culture within the school building that is supportive of student mental health. For example, the skills and behaviors of leaders are frequently emphasized as critical drivers of organizational impact across various workplaces, including schools (Stadnick et al., 2019; Suhrheinrich et al., 2021). Outcome studies of effective school mental health programs also highlight the importance of leadership implementation strategies that produce high levels of adoption and use of evidence-based practices (Lyon et al., 2022). Although many school and community personnel are responsible for implementation, school leaders are often seen as pivotal in this process because their promotion of buy-in is associated with whether, and to what extent, mental health promotion and prevention develop and are sustained in school contexts (Gottfredson & Gottfredson, 2001; Kam et al., 2003).

While acknowledging that implementation leadership behaviors may vary significantly across contexts, Lyon and colleagues (Lyon et al., 2022) argue that strategic implementation leadership is an important component of successful leadership and a key determinant of successful implementation of school mental health promotion, prevention, and intervention. Behaviors associated with strategic implementation leadership include strategic communication and direct support of staff or personnel to achieve a strategic goal for new practices and programs (Schein, 2010), as well as holding staff and personnel accountable for achieving implementation goals (Lyon et al., 2022). In a recent study that sought to adapt and validate the School Implementation Leadership Scale (Lyon et al., 2022), results supported the relevance of the original subscales including being proactive, knowledgeable, supportive, and perseverant. Additional new subscales that emerged included communication, vision/mission, and availability. As described by the authors, communication by school leaders involves efforts to engage in bidirectional communication surrounding the implementation of evidence-based practices; vision/mission comprises how well the school leader embeds the implementation of evidence-based practices into the core objectives of a school; and availability reflects leaders being accessible and responsive to staff needs or problems pertaining to implementation (Lyon et al., 2022).

School leaders play a key role in promoting and influencing school climate (Hallinger & Heck, 1996), which is associated with the implementation and outcomes of mental health prevention and intervention programs (Langley et al., 2010). This influence is in part due to their impact on school professionals (e.g., teachers, counselors) who are most likely to be involved in the day-to-day implementation of activities that support student mental health. For example, teachers are well positioned to successfully implement mental health promotion and intervention efforts using evidence-based programming, positive teacher–student interactions, and embedded lessons in the curricula (Weist, 2005). Teacher participation is reliant on school climate, including principal attitudes, beliefs, knowledge, and general involvement and support, which are crucial for successful school mental health promotion (Dadaczynski et al., 2022). For example, consistent support from school leaders for school professionals is needed to cultivate effective practices as teachers’ own social and emotional competence has been found to influence the implementation of social and emotional learning practices (Reyes et al., 2012). Guskey and Sparks (1996) suggest that school leaders indirectly influence students through interactions with teachers by contributing to school climate, school culture, and dedication to continuous improvement. A study by Hudson and colleagues (Hudson et al., 2020) found that schools were more successful in implementing mental health interventions when the school leaders were more involved and actively engaged in supporting the intervention. Ekornes (2015) also found that limited or weak support from school leadership often resulted in poor integration of school mental health promotion into school routines and policy.

### 1.3. Models and Best Practices for Leadership Preparation Programs and Professional Development

To realize the full potential of school leaders in supporting student mental health, individual and organizational leadership behaviors can be enhanced through effective leadership preparation programs and professional development. This section broadly reviews the existing research on promoting effective leadership practices as these strategies and models may be adapted for leaders interested in or committed to promoting mental health in their schools. The current contribution then adds to this literature by highlighting examples of leadership preparation and professional development programs designed specifically to support school mental health promotion (see Table S2).

Leadership preparation programs are considered preservice opportunities that occur before a school leader enters the leadership role, while professional development typically refers to learning opportunities that occur once a leader is on the job. Interest in school leadership preparation programs outside North America did not materialize prior to the mid-1990s (Hallinger, 2003), although there is an increasing acknowledgment that the professional development of school leaders is essential to help them navigate the complex challenges they encounter daily (Mahfouz et al., 2019). However, educational leadership preparation programs rarely include content that addresses the mental health of students, field experiences that highlight effective school mental health programs, or well-defined and measurable expectations of school principals to prepare them for the realities of addressing the mental health challenges experienced by some segments of youth in their school. The research on the effectiveness and outcomes of different leadership preparation models is limited, but several descriptive studies have identified key components and essential characteristics of programs considered exemplary in preparing early career professionals to become effective school leaders. As Cunningham and colleagues (Cunningham et al., 2018) describe, exemplary leadership preparation programs include components that promote the development of three types of knowledge: (1) declarative (i.e., ability to describe effective leadership principles), (2) procedural (i.e., ability to implement leadership skills), and (3) contextual (i.e., ability to appropriately match leadership actions to specific contexts or situations). Recent efforts have been undertaken to review and synthesize the past two decades of the literature on leadership preparation programs. In one review, the authors sought to better understand the evidence regarding high-quality principal learning, with a specific focus on examining the features of principal preparation and development programs and their relationship to principal, teacher, and student outcomes (Darling-Hammond et al., 2022). Another literature review focused on synthesizing results from high-quality, rigorous research designs that examined professional development programs (Donley et al., 2021). Findings from these respective reviews reveal several core features that are commonly used across effective training models to promote the development of these three levels of knowledge (Darling-Hammond et al., 2022; Donley et al., 2020). These key program features can be summarized in the following categories: (1) comprehensive, standards-based curricula emphasizing instructional leadership, (2) applied clinical experiences (e.g., field-based internships or residencies) with adequate mentoring or coaching, and (3) cohort models or program structures conducive to collegial partnerships (Darling-Hammond et al., 2022; Donley et al., 2020).

## 2. Comprehensive Curricula Emphasizing Instructional Leadership

Findings from the literature review by Donley and colleagues (Donley et al., 2020) indicated that a core component of exemplary leadership preparation programs is their use of comprehensive curricula designed to equip candidates with knowledge and skills for instructional leadership, change management, problem-solving, organizational management, cultural competence, and community engagement. Historically, preparation programs for prospective leaders were largely focused on administrative aspects of school management, such as budgeting (McCarthy, 2015). While competency in organizational management (i.e., knowledge of fiscal management, resource plans, ethical and legal regulations, etc.) remains one of the key components of effective leadership preparation programs (Hudson et al., 2020), a growing emphasis on using curricula to teach instructional leadership has emerged as an important aspect of the national standards for leadership preparation programs (Hackmann & McCarthy, 2011).

The National Policy Board for Educational Administration (NPBEA)’s National Educational Leadership Preparation (NELP) Program Recognition Standards emphasize instructional leadership, or “the knowledge, skills, and commitments a leader needs to diagnose, develop, implement, and evaluate coherent systems of curricula, instruction, data systems, supports, and assessment”, as a central competency for school leaders (NPBEA, 2018, p. 88). Instructional leadership training encompasses not only the skills prospective leaders will use to develop and evaluate systems to support the diverse educational needs of their students but also essential leadership skills for facilitating collaboration with teachers and other school staff members to encourage shared decision-making and support staff development (Darling-Hammond et al., 2022). Some exemplary programs, such as the University of Connecticut’s Administrator Preparation Program, include explicit opportunities for leaders to develop this skillset through collaborative leadership assignments wherein they practice actively encouraging teacher input in decision-making processes (Darling-Hammond et al., 2007).

Many exemplary principal preparation programs also include in their curricula an emphasis on both qualitative and quantitative data-driven frameworks leaders can utilize to identify problems and develop and enact school improvement plans (Hudson et al., 2020; Sutcher et al., 2017). For example, as described by Cosner and colleagues (Cosner et al., 2015), the Urban Education Leadership principal preparation program through the University of Chicago trains leaders to adopt a data-driven approach to problem-solving by using a “cycle of inquiry” model (Cosner et al., 2015). This model involves identifying the root causes of the problem, planning for the enactment of specific remedial strategies to address the root causes of the problem, operationalizing outcome goals and establishing goal assessment plans, enacting strategy action plans, assessing progress toward goal outcomes, and diagnosing and implementing necessary adjustments (Cosner et al., 2015; Donley et al., 2020).

Another critical component of effective leadership preparation program curricula is an emphasis on cultural competence and social justice to prepare school leaders to equitably and inclusively address the needs of a diverse community of learners (Darling-Hammond et al., 2022). According to Darling-Hammond and colleagues (Darling-Hammond et al., 2022), research suggests that including as few as one course on diversity, equity, and inclusion can increase leaders’ sense of cultural responsiveness. It is important to recognize that didactic training alone is insufficient in instilling the skills necessary for effectively leading multiculturally diverse school communities. Development in this area requires a combination of coursework and applied experience working directly within these student communities to promote equitable learning opportunities (Cosner et al., 2015). The School District of Philadelphia’s Leadership Pathways Framework (2022) exemplifies these values through their core emphasis on equity-centered leadership, which aims to “cultivate prosperity and liberation for all staff and students, starting with historically marginalized populations, by applying an equity lens to all leadership situations and interactions”. (School District of Philadelphia, 2022) This program defines key skills and behavioral indicators within this domain that leaders are trained to enact, including (1) setting equitable expectations for all students; (2) increasing access and inclusion by implementing systems and support structures that are inclusive of diverse backgrounds and strengths; (3) facilitating and building trusting relationships with families and community members by amplifying historically marginalized voices; (4) removing structural barriers by engaging in critical self-reflection and dismantling inequitable school policies; and (5) creating a culture of shared social responsibility focused on continuous growth and learning (School District of Philadelphia, 2022). Although the literature from the past two decades reflects an emerging emphasis on addressing equity and inclusion concerns in high-quality leadership preparation programs, further measures are required to improve access to these exemplary programs by leaders who seek to work in socioeconomically marginalized school communities (Darling-Hammond et al., 2022).

## 3. Applied Learning Opportunities with Mentoring

Exemplary leadership preparation programs also include opportunities for candidates to develop procedural knowledge by integrating theory and practice through active engagement in applied learning opportunities. Field-based clinical internships or residencies provide invaluable opportunities for candidates to refine their leadership and administrative skills through direct clinical service in the school community, under the supervision and mentorship of expert leaders (Donley et al., 2020). Research suggests that internships are a key component of preparation programs, with graduates of programs that include strong internship opportunities reporting higher self-efficacy and perceived preparedness for the field (Orr & Barber, 2006; Versland, 2016). Despite these findings, high-quality internship programs that allow candidates to engage meaningfully in authentic leadership tasks are less readily available (Darling-Hammond et al., 2022). In fact, only 46% of principals endorsed having an internship that allowed them to take on authentic leadership roles, and fewer still reported having access to mentoring or coaching during the internship (Darling-Hammond et al., 2022).

Individualized mentorship or coaching by an experienced school leader is a crucial aspect of effective leadership preparation programs, allowing for prospective leaders to learn from their mentor’s effective modeling of leadership skills (Sutcher et al., 2017; Zepeda, 2013). Research suggests that proximity of the mentor (i.e., sharing the same building; ability to meet face-to-face), shared priorities, and trust are key elements of successful clinical training experiences for prospective leaders (Clayton & Myran, 2013; Donley et al., 2020). Despite evidence that mentorship helps strengthen prospective leaders’ capacity for effective leadership, there remains significant inequity in access to high-quality mentorship experiences, with leaders serving in high-poverty schools less than half as likely than those serving in low-poverty schools to have access to an on-site mentor or coach (Darling-Hammond et al., 2022).

Other applied learning experiences, such as case study analyses, problem-based activities, and simulations or role plays, have been found to strengthen prospective leaders’ procedural and contextual knowledge of leadership skills (Donley et al., 2020). For example, the University of Connecticut’s Administrator Preparation Program utilizes “SchoolSims”, a variety of simulations that mimic real-world leadership situations and provide the opportunity for candidates to engage in active problem-solving, implement a chosen course of action, and experience the consequences of their decision (UCAPP, n.d.). Other programs, such as the Educational Leadership Program for Aspiring Principals at the University of Pennsylvania (Donley et al., 2020), include required school visits wherein prospective leaders visit classrooms to observe teachers and students and review the curricula (Darling-Hammond et al., 2022). A study by Brody and colleagues (Brody et al., 2010) suggests that leadership preparation programs that include a direct classroom observation component may equip prospective leaders with a vision of their role as leaders to embrace critical inquiry and understand the complex nature of organizational change.

## 4. Cohort Models and Partnership Structures

Cohort models, whereby candidates enter and progress through leadership preparation programs together, represent another key component of high-quality training programs (McCarthy, 2015). According to a review by Donley and colleagues (Donley et al., 2020), these models are associated with several positive outcomes, including increased likelihood of program completion (Nimer, 2009), improved capacity for prospective leaders to develop long-standing peer relationships and networks, and strengthened sense of trust in their programs (Salazar et al., 2013). Overall, the cohort program structure is conducive to collegial learning, including opportunities for shared reflection, peer coaching opportunities, and shared recruitment and curriculum design efforts through the development of leadership networks (Darling-Hammond et al., 2007; Donley et al., 2020).

Program-district partnerships between universities that house leadership preparation programs and school districts are an effective way to promote collaboration and enable candidates to more seamlessly bridge their theoretical knowledge from coursework to clinical practice (Mendels, 2016; Sanzo et al., 2011). Through these partnerships, university faculty from leadership preparation programs and key school district personnel jointly conduct candidate recruitment and selection for preparation programs and engage as a team to collaboratively provide instruction to candidates (Donley et al., 2020; Sutcher et al., 2017). While promising, these partnerships are rare and would benefit from further support through policy change efforts (Donley et al., 2020; Sanzo et al., 2011).

### School Leadership Training Programs to Support School Mental Health Promotion

There is limited research related to the specific and most effective leadership behaviors, skills, and competencies for supporting the mental health of students, including through the implementation of school-wide mental health promotion programs. This section describes a case example of a leadership training program focused on developing a comprehensive school mental health system, a call to action for school leaders to integrate health (and mental health) into professional development programs, and an evaluation study of professional development workshops whose objective was to support leaders’ capacity to improve student social, emotional, and behavioral health outcomes.

The Massachusetts Partnerships for Youth, Inc. ran a School Mental Health Leadership Institute in 2023 whose objective was to prepare leaders to drive change within their school or district, with the goal of supporting student well-being. Program content involved lessons on conducting a needs assessment, establishing a comprehensive student support team, implementing tiered interventions, gathering and using data, and engaging in continuous improvement. The sessions comprised ten hours of professional development and training with the opportunity to apply and practice learning in between sessions. Strengths of this professional development program include a cohort-based model, a virtual learning platform, and planned meetings for school and district leaders to share their learning and best practice strategies for advancing a comprehensive school mental health system. The advantages of these types of programs are that they bring together a “fellowship of the willing” and cultivate leaders who are committed to the hard work involved in developing, implementing, maintaining, and hopefully scaling school-wide mental health and not just single intervention programs.

In a recent call to action article, Leksy and colleagues (Leksy et al., 2024) highlighted the lack of health promotion in preservice training and professional development for school leaders and noted that little research exists around which leadership competencies are needed and to what extent they are taught. Dadaczynski and colleagues (Dadaczynski et al., 2022) proposed three perspectives of leadership in school health promotion, although these same perspectives can easily be applied to school mental health promotion: self-related health-promoting leadership (school principals who support their own health needs), staff-related health-promoting leadership (school principals who pursue health promotion for school staff and demonstrate interest in work’s influence on satisfaction and well-being), and intervention-related health-promoting leadership (management practices and activities to support health-promotion change processes in school). The three perspectives and dimensions of principal-supported school health promotion can largely influence school conditions for staff and students.

School leaders play a primary role in decision-making and are, therefore, instrumental in initiating, implementing, and sustaining mental health promotion efforts and integrating them into the school organization and culture (Leksy et al., 2024). When the proposed perspectives by Dadaczynski and colleagues (Dadaczynski et al., 2022) are adapted for mental health, school leaders would participate in preservice and professional development training to learn about managing and protecting their own mental health, providing care and resources that support the mental health of teachers and staff at the school, and to developing engagement strategies to support important aspects of implementation activities for school mental health promotion.

Kaye and colleagues (Kaye et al., 2022) evaluated professional development workshops facilitated by school-based behavioral health providers and delivered to various school professionals, including school administrators (e.g., principals) to strengthen staff capacities around the promotion of social, emotional, and behavioral health of students. Professional development workshops were designed within the context of a learning collaborative model and topics included (1) an overview of social and emotional development to inform appropriate expectations; (2) strategies for supporting students in the classroom; (3) information on behavioral health, symptoms, and systems; (4) tips and tools for classroom management; (5) stress management and self-care for the educator; (6) trauma-informed conceptualization to learning; (7) developing strategies for addressing social and emotional health and implementing school-wide initiatives; (8) building effective teams; (9) cultivating engaged partnerships with families; (10) strategizing for sustainability; and (11) managing transitions. These workshops utilized a combination of didactic presentation and discussion, surrounding content application to participant’s specific schools, and opportunities to discuss goals and action steps. In response to research highlighting the importance of supporting school professionals’ own social and emotional competencies (Jennings & Greenberg, 2009), an emphasis on self-reflection was also built into the workshops. In alignment with the objectives of the workshops, findings revealed increases in school professionals’ knowledge, skills, and self-efficacy relevant to student social, emotional, and behavioral health (Jennings & Greenberg, 2009). Participants also reported satisfaction with the professional development workshops, indicating an ability to apply learnings and implementation strategies to their individual schools (Jennings & Greenberg, 2009). Strengths of this program include school-based behavioral health providers serving as workshop facilitators, the use of a learning collaborative model, and mapping content to align with the context of individual schools.

Another valuable resource that can support engagement and implementation activities for school mental health promotion is Classroom Well-Being and Information for Educators (WISE), a free, three-part mental health literacy training package for educators and school staff that includes an online course, video library, and resource collection (Semchuk et al., 2022). The objective of this training package is to provide opportunities for education, training, and reflection focused on topics such as general mental health promotion content, including how to create welcoming and supportive classrooms, how to infuse mental health literacy into the curriculum, and how to foster social and emotional competencies of students and staff. Modules from WISE also include content specific to identifying and supporting students exposed to adversity or those struggling with psychological distress (Semchuk et al., 2022). The developers of WISE sought to address often-cited barriers for educators and school leaders by making the program free, self-paced, relatively short (about 5 h per trainee), and always accessible (available 24/7) (Semchuk et al., 2022). It is important to highlight that although Classroom WISE includes evidence-based strategies to promote student mental health and support students with mental health challenges, to date, there are no published studies that evaluate outcomes for students and/or teachers following educator or school personnel participation in this course.

## 5. Conclusions and Future Directions

School leaders have the influence to promote or discourage almost any initiatives started at their respective schools, including the implementation of best practices to address the mental health needs of students. However, school leaders also must balance this responsibility with increased pressure to focus on academics and make up for delayed learning caused by the pandemic. In the current post-pandemic context where symptoms of mental health issues have significantly increased among youth, effectively addressing the mental health needs of students represents a complex and critical challenge for school leaders. A review of the research did not reveal any systematic reviews or meta-analyses that investigated skills or competencies needed by school leaders to most effectively support student’s mental health. Instead, available research on supporting student’s mental health has primarily focused on the effectiveness or efficacy of mental health interventions at the individual, group, or school level. While this research is critical, it often fails to evaluate how school leaders can play a crucial role in supporting student mental health vis-à-vis selecting evidence-based interventions (EBI) (Moore et al., 2024) and designing organizational influences and structures that support the successful implementation of these EBIs (Lyon et al., 2022). Promisingly, there are recent efforts in the implementation science literature to investigate how school leaders understand evidence associated with selecting EBIs to adopt to their local school context (Walker et al., 2022).

Another research priority for promoting effective leadership in schools related to supporting student mental health is identifying the skills and competencies school leaders need to collect and analyze data about students’ mental health and well-being. Scholars have recently emphasized the importance of school leadership in partnering with stakeholders to design or select progress monitoring and interconnected data systems (Weist et al., 2023; Connors et al., 2022). It also has been suggested that data collection and measurement in school mental health should move beyond tracking mental illness indicators to include measures of well-being given that so much of the focus in schools is on mental health promotion and strength-based programming (Weist et al., 2023; Connors et al., 2022).

**Figure 1:**
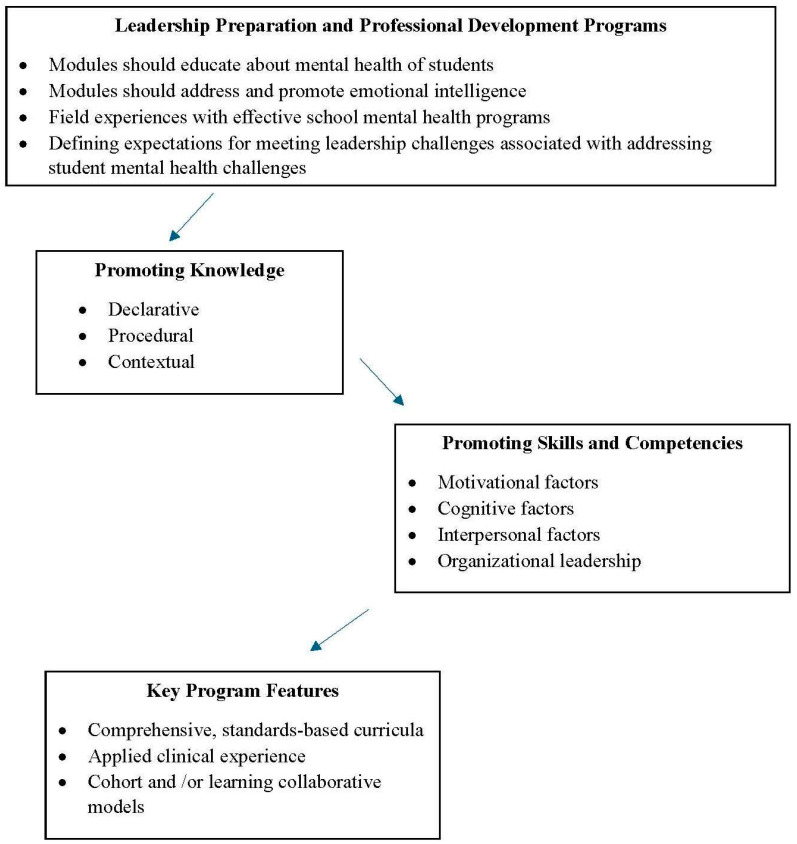
Key considerations when designing an effective leadership training program.

In 2006, Koller and Bertel commented, “Therefore, there is a call for a paradigm shift at the preservice level to better prepare school administrators to confront proactively the mental health challenges of today’s youth and the difficulties they face in serving those students” (Koller & Bertel, 2006). This call to action was made in 2006, yet little progress has been made toward developing and empirically evaluating training and professional development programs focused on supporting student mental health delivered to aspiring and current school leaders, especially with a focus on the long-term effectiveness of these programs (Patti et al., 2015). The design of preservice programs for aspiring school leaders and professional development programs for current school leaders should be informed by previous findings related to effective leadership behaviors, skills, and competencies. For example, there is some evidence to support programs focusing on enhancing motivational factors, cognitive factors, and interpersonal factors to improve leadership behaviors (Newlove-Delgado et al., 2021). In particular, the skills/competencies of self-awareness, self-management, and empathy (Boyatzis et al., 2000) should be targets of intervention in these programs given the association between these skills and effective leadership. In addition to individual leadership behaviors, training and development programs should include content and practice opportunities for organizational leadership behaviors such as strategic implementation leadership behaviors (Lyon et al., 2022), including a focus on best practices for promoting a positive school climate and culture (see Figure 1).

Although it is well established that school leaders are instrumental in supporting students’ positive mental health (Short, 2016), prior to the pandemic, this responsibility was often delegated to school mental health professionals with little involvement or less oversight by the school leader. As the rates of student mental health problems have increased since the COVID-19 pandemic, school leaders increasingly recognize the centrality of their leadership in supporting the socio-emotional and mental health of students in their schools (Adams & Olsen, 2017). However, there is limited research related to the effectiveness of leadership training and professional development programs for school leaders who want to improve school-wide mental health. It is possible that more programs are being developed that provide leadership training for supporting student mental health, but the accompanying empirical studies have yet to be published. It also may be true that supporting student mental health is included in broader leadership training programs but represents a smaller component. Programs such as WISE are promising because the design is responsive to barriers cited by busy school leaders (e.g., time commitment and easy access to content); however, important best practice elements are missing such as applied learning opportunities with mentoring and cohort program structures that promote collegial learning.

This narrative literature review found a lack of control groups in the available studies, which is important for better understanding the relationship between the training and development program and outcomes for student mental health and well-being. Moreover, there are few studies that compare different training approaches to determine which types of programs work best across different types and settings of schools. For example, our review did not find any training approaches or programs that sought to tailor instruction in leadership development based on challenges encountered in under-resourced schools located in disadvantaged areas compared to more resourced schools located in affluent neighborhoods. Therefore, there is a need for future studies that include control groups as well as comparison studies for schools with different demographics and that evaluate the impact of various design elements of the programs such as standards-based curricula, emphasizing instructional leadership, applied clinical experiences with adequate mentoring or coaching, and cohort models or program structures conducive to collegial partnerships (Darling-Hammond et al., 2022; Donley et al., 2020). When control group studies are not feasible due to practical limitations, such as limited resources or a small sample size, quasi-experimental studies can be helpful in understanding the effect of the intervention on that group of school leaders, including the adoption of leadership behaviors, skills, and competencies.

We know that effective leadership is crucial for successfully promoting student mental health in schools. Although there have been some smaller-scale efforts, the fields of education and psychology must come together to design, evaluate, improve, and scale preservice and professional development programs that are responsive to enhancing leadership behaviors and that connect these behaviors to students’ social and emotional outcomes.

## Data Availability

The original contributions presented in this study are included in the article/Supplementary Materials. Further inquiries can be directed to the corresponding author.

## Supplementary Materials

The following supporting information can be downloaded at: https://www.mdpi.com/article/10.3390/bs15010036/s1. Table S1. Skills, Competencies, and Behaviors for School Leaders Supporting Student Mental Health. Table S2. Models and Best-practices for Leadership Preparation Programs and Professional Development.
