# Supplementary material for: Effective School Leadership for Supporting Students’ Mental Health: Findings from a Narrative Literature Review
Source: Behav Sci (Basel). 2025 Jan 1;15(1):36. doi: 10.3390/bs15010036 (PMC11761599; doi:10.3390/bs15010036)
Supplement: Supplementary file 1 [file behavsci-15-00036-s001.zip › Table S1.pdf]

Table S1.

Skills, Competencies, and Behaviors for School Leaders Supporting Student Mental Health

Leadership Domains	Skills, Competencies, Behaviors	Resources	Qualitative rating[1]
Effective School Leadership - General	Time management, influence, decision-making, commitment, communication	1 Sun, H.; Wang, X.; Sharma, S. A Study on Effective Principal Leadership Factors in China. *International Journal of Educational Management* 2014, *28* (6), 716–727. https://doi.org/10.1108/ijem-11-2013-0173.	Evidence-based practice
Effective School Leadership – Student Mental Health	Social and emotional competencies; emotional intelligence	1 Gómez-Leal, R.; Holzer, A. A.; Bradley, C.; Fernández-Berrocal, P.; Patti, J. The Relationship between Emotional Intelligence and Leadership in School Leaders: A Systematic Review. *Cambridge Journal of Education* 2021, *52* (1), 1–21. https://doi.org/10.1080/0305764X.2021.1927987.<br>2 Sánchez-Núñez, M.; Patti, J.; Holzer, A. (2015). Effectiveness of a leadership development program that incorporates social and emotional intelligence for aspiring school leaders. *Journal of Educational Issues* 2015, *1*(1), 65-84. 10.5296/jei.v1i1.	Research-based practice
Strategic Implementation Leadership – Organizational Factors	Strategic communication and direct support of staff or personnel; accountability for implementation goals; bidirectional communication regarding implementation;	1. Lyon, A. R.; Corbin, C. M.; Brown, E. C.; Ehrhart, M. G.; Locke, J.; Davis, C.; Picozzi, E.; Aarons, G. A.; Cook, C. R. Leading the Charge in the Education Sector: Development and Validation of the School Implementation Leadership Scale (SILS). *Implementation Science* 2022, *17* (1). https://doi.org/10.1186/s13012-022-01222-7.<br>2. Langley, A. K.; Nadeem, E.; Kataoka, S. H.; Stein, B. D.; Jaycox, L. H. Evidence-Based Mental Health Programs in Schools: Barriers and Facilitators of Successful Implementation. *School Mental Health*	Promising practice

	determining a vision/mission; leader availability	2010, *2* (3), 105–113. https://doi.org/10.1007/s12310-010-9038-1.	

Note[1]: Qualitative ratings for the extent of evidence that currently supports each skill/competency/behavior is based on those from the IRIS Center of Vanderbilt University: https://iris.peabody.vanderbilt.edu/module/ebp_01/cresource/q1/p01/
