# Supplementary material for: Effective School Leadership for Supporting Students’ Mental Health: Findings from a Narrative Literature Review
Source: Behav Sci (Basel). 2025 Jan 1;15(1):36. doi: 10.3390/bs15010036 (PMC11761599; doi:10.3390/bs15010036)
Supplement: Supplementary file 1 [file behavsci-15-00036-s001.zip › Table S2.pdf]

Table S2.

Models and Best-practices for Leadership Preparation Programs and Professional Development

Program Domains	Program Goals	Resources
Comprehensive Curricula Emphasizing Instructional Leadership	Knowledge and skills for instructional leadership, change management and problem solving, organizational management, cultural competence, and community engagement	1 National Policy Board for Educational Administration. *National Educational Leadership Preparation (NELP) Program recognition standards: Building level.* 2018. http://www.npbea.org/wp-content/uploads/2018/11/NELP-Building-Standards.pdf (accessed August 8, 2024).<br>2 Darling-Hammond, L.; LaPointe, M.; Meyerson, D.; Orr, M. T.; Cohen, C. Preparing School Leaders for a Changing World: Lessons from Exemplary Leadership Development Programs. School Leadership Study. Final Report. *Stanford Educational Leadership Institute.* 2007.<br>3 Sutcher, L.; Podolsky, A.; Espinoza, D. Supporting Principals' learning: Key features of effective programs. *Learning Policy Institute* 2017.
Applied Learning Opportunities with Mentoring	Develop procedural knowledge by integrating theory and practice through active engagement in applied learning opportunities	1 Orr, M. T.; Barber, M. E. Collaborative Leadership Preparation: A Comparative Study of Partnership and Conventional Programs and Practices. *Journal of School Leadership* **2006**, *16* (6), 709–739. https://doi.org/10.1177/105268460601600603.<br>2 Versland, T. M. Exploring self-efficacy in education leadership programs: What makes the difference? *Journal of Research on Leadership Education* **2016**, *11*(3), 298–320. https://doi.org/ 10.1177/1942775115618503
Cohort Models and Partnership Structures	Opportunities for collegial learning, including shared reflection, peer coaching, and shared recruitment and curricula design efforts through development of leadership networks	1. Darling-Hammond, L.; LaPointe, M.; Meyerson, D.; Orr, M. T.; Cohen, C. Preparing School Leaders for a Changing World: Lessons from Exemplary Leadership Development Programs. School Leadership Study. Final Report. *Stanford Educational Leadership Institute.* 2007.
Student Mental Health Promotion	Support school leaders' capacity to improve student social, emotional, and behavioral health outcomes	1. Leksy, K.; Gawron, G.; Rosário, R.; Marjorita Sormunen; Velasco, V.; Sandmeier, A.; Venka Simovska; Wojtasik, T.; Dadaczynski, K. The Importance of School Leaders in School Health Promotion. A European Call for Systematic Integration of Health in

		Professional Development. *Frontiers in Public Health* **2024**, *4* (11). https://doi.org/10.3389/fpubh.2023.1297970.<br>2. Kaye, A. J.; Pejic, V.; Moffa, K.; Jordan, M.; Dennery, K. M.; DeMaso, D. R. Using Professional Development Workshops to Support School Professionals' Capacities to Promote Students' Social, Emotional, and Behavioral Health. *Psychology in the Schools* **2022**, *59* (4), 866–880. https://doi.org/10.1002/pits.22652.<br>3. Semchuk, J. C.; McCullough, S. L.; Lever, N. A.; Gotham, H. J.; Gonzalez, J. E.; Hoover, S. A. Educator-Informed Development of a Mental Health Literacy Course for School Staff: Classroom Well-Being Information and Strategies for Educators (Classroom WISE).
